# Taming the torrent: changes in flood protection at the Gürbe River (Switzerland) from the nineteenth century until today

**DOI:** 10.1007/s12685-022-00312-z

**Published:** 2022-11-17

**Authors:** Melanie Salvisberg

**Affiliations:** Section of Economic, Social and Environmental History, Institute of History & Oeschger Centre for Climate Change Research, University of Bern, Länggassstrasse 49, 3012 Bern, Switzerland

**Keywords:** Environmental history, Historical hydrology, Flood protection, Mountain torrent, Infrastructure, Revitalisation

## Abstract

This paper analyses the flood protection history of the Gürbe River (Switzerland), a 29-km-long tributary of the Aare River. The upper reach of the river has the character of a mountain torrent and an exceptionally difficult flooding situation. For centuries, riparian communities were only able to take small protective measures. In the mid-nineteenth century, the flood protection strategy changed: between 1855 and 1881, the Gürbe River was channelised and stabilised by a torrent control system. Although the situation improved, flood damage could not be prevented as intended. Therefore, dozens of consecutive projects were implemented—without interruption until today. This paper examines why small watercourses are useful case studies, which protection measures were taken at the Gürbe River, how they corresponded to the prevailing flood protection philosophy, whether they were linked to floods and how flood protection influenced land use. The Gürbe regulation, its consecutive projects and the connected drainages had far-reaching effects: They allowed an intensive agricultural use of the valley floor, the construction of roads, a railway, and new settlements. Consequently, the social and economic pressure on the hazard area increased steadily over the decades. It created a vicious circle: the more that protective structures were built, the more important and profitable flood prevention became, and the more structures were raised. A reevaluation finally took place in the late twentieth century, based on increasing environmental awareness, and fostered by a catastrophic flood. However, the implementation of new projects proved to be difficult due to conflicting interests.

## Introduction

Today, natural rivers have become a rarity in western and central Europe. In Switzerland—a country rich in water bodies—the situation is particularly bad: 95% of the rivers and streams are embanked, most of them from the source to the mouth (Koblet 1995; WWF 2016). The process leading to this situation started in the nineteenth century. Against a background of increased demand for land for the growing population, new technologies, severe floods, and the industrialisation process, people started to modify the watercourses. Firstly, the large rivers were channelised, and later also the small streams and mountain torrents. These interventions into nature had far-reaching effects on land use, settlement, and transport development, since they created a self-reinforcing process: the more the rivers were modified and the better the valleys were protected, the more intense the land use and the stronger the requests for further corrections and flood prevention measures became (Di Baldassarre et al. 2013). This cycle was not questioned for decades, and the various unintended effects such as the ecological consequences were not discussed. Reevaluations have taken place only since the late twentieth century, but the implementation of improvement measures, such as renaturatlization, proved to be difficult.

Information about past floods is indispensable for those living or building around bodies of water. In Switzerland, more than 1.1 million people (13% of the population) live in flood risk areas, and about 270,000 buildings are endangered. The alpine and pre-alpine regions are particularly affected (Mosimann et al. 2017). Long-term knowledge on floods is not only useful for detecting the causes, frequencies, and gravity of floods, but also for understanding their natural variability and changes over time (cf. Merz et al. 2014; Seneviratne et al. 2014; Wilhelm et al. 2018). Furthermore, it helps improve awareness of the flood risk (Grünewald 2010).

This paper aims to discuss the modification of small rivers and the changes in flood protection on the Gürbe River, a tributary of the Aare River. In its upper reach, the Gürbe has the characteristics of a mountain torrent, making flood protection particularly difficult. This article addresses the following questions: (1) Why are small watercourses useful case studies on historical flood prevention measures? (2) What kinds of flood protection measures were implemented on the Gürbe River, and did they correspond to the prevailing protection philosophy? (3) How were protection measures connected to flood events? (4) How has flood protection on mountain torrents changed in the last two decades? (5) How did flood protection measures impact land use?

Information about the occurrence of historical floods, their seasonal distribution, or their causes provides knowledge about the changes in frequencies and intensity of floods, and it helps us to analyze flood risks. Small watercourses in particular are highly suited for studies on historical floods and flood protection for several reasons: compared to large rivers, small rivers and creeks respond more directly to precipitation. Furthermore, they are more sensitive to changes on the watercourse and the surrounding area. From a historian’s perspective, studies on a small river have substantial advantages. The limited spatial extent allows long-term studies and deeper analyses on the interdependences between flood protection measures and natural processes. This is particularly true for the Gürbe River which is highly prone to floods due to the hydrological and geological conditions. Despite best efforts the floods could never be prevented, which is the reason why the flood protection associations implemented big hydraulic engineering projects without interruption from 1855 until today. Additionally, research materials are available in large numbers since the flood protection stakeholders such as the local flood protection cooperatives (consisting of delegates of the landowners and municipalities) and cantonal and federal hydraulic engineers provided their documents and shared their knowledge. Combined with the records of the public archives this resulted in an unusually broad and large body of sources.

Despite the advantages of small watercourses for longitudinal studies, historical research has so far mostly focused on the large rivers, especially in densely populated areas (e.g., Bernhardt 2016; Bronar Cafaro 2004; Cioc 2002; Lewis 2005; Lübken 2014; Reynard 2009; White 1995). Flood protection measures on medium and small rivers have been researched, by, among others, Armenat (2012), Deutsch (2007), Heinzmann (2019), Hügli (2007) and Speich (2003). Himmelsbach (2014) analysed and compared the historical floods and flood protection measures on 15 non-navigable tributaries of the upper Rhine River. Studies on the historical flood protection measures on alpine rivers were published by Girel (2008), Gurnell et al. (2009), Hauer et al. (2019) and Hohensinner et al. (2020). Mountain torrents have been an important research object of hydraulic engineering sciences in the last decades since they face major challenges such as the overageing of the existing protective structures (see Stauder 2014). However, the historical perspective has remained sparse: Piton et al. (2016) discussed the French experience of 150 years of torrent control works and compared it to other countries. Göttle (1996) presented an overview over the last hundred years of torrent control in Bavaria, Aulitzky (1994) and Patek (2008) examined the historical torrent control in Austria, Blinkov et al. (2013) in the Balkans, and Jakubis and Jakubisová in Slovakia (2019). Case studies were conducted, amongst others, by Egloff (2016) and Keller (2013).

## The Gürbe River

With its length of only 29 km and a catchment area of 131 km^2^, the Gürbe River is a small watercourse which looks harmless on most days. But this can change within a few hours: after heavy rainfalls the water swells rapidly and the rivulet turns into a raging river with the potential to cause substantial damage. Despite its small size, the Gürbe River must be divided into two parts: the ten-kilometre-long upper reach in the southern, pre-Alpine region is characterized by its steep slope (on average 12.9 degrees, respectively 23%) while the lower reach enters the Swiss midlands and is flat (0.09–0.23 degrees, resp. 0.15–0.4%).

After its source at an altitude of 1685 m above sea level on the Alp Obernünenen in the Gantrisch region, the water flows as a small rivulet through the conical headwaters for a few hundred metres. Afterwards, the Gürbe River runs through a steep gorge. Due to the soft rock in this area (predominantly flysch and molasse), the river and its tributaries cause huge amounts of bed load that the river discharges during high water since flysch is easily eroded and water impermeable. Moreover, the area is highly unstable and therefore prone to landslides. After the gorge, the slope lowers significantly, and the water deposits the transported debris on a large alluvial fan. With the steep slope, the small catchment area of only 12.1 km^2^, the strongly varying runoff and the temporarily high sediment discharge, the upper reach of the Gürbe River fulfils the criteria of a mountain torrent (cf. Loat and Meier 2003).

The end of this transition zone marks the beginning of the lower reach of the Gürbe River. From here, the river flows about 20 km through the flat valley floor. The valley opens in the area of Belp, where the Gürbe River finally enters the Aare River. The tributaries in the lower reach are smaller and less dangerous than the torrents in the headwater, but these affluents can cause considerable damage since they swell quickly after heavy rainfall.

**Fig. 1:**
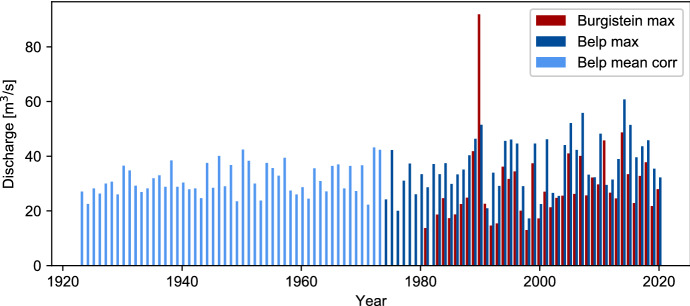
Yearly maximum discharge measurement from measurement stations in Burgistein (red) and in Belp (light blue and dark blue). Before 1974 only daily mean discharge values are available for the Belp. These data are corrected (light blue) using a linear regression of data from 1974 to 2020, where both daily maximum and daily mean discharge values are available (Q_max_ = 14.6 [m^3^/s] + 1.109 × Q_mean_, R_Pearson_ = 0.793). Data Belp: BAFU 2021, data Burgistein: AWA (2021)

The long-term annual mean discharge of the Gürbe River is 1.33 m^3^/s in the upper reach (Burgistein) and 2.62 m^3^/s in the lower reach (Belp) (measuring period 1923–2020 resp. 1982–2020). As is typical for a mountain torrent, the discharge fluctuates heavily. The highest daily maximum discharge measured in Burgistein is 91.9 m^3^/s (29th Juli 1990), while the lowest is 0.13 m^3^/s (30th October 1985), the ones in Belp amount to 60.8 m^3^/s on 11th August 2014 resp. 0.15 m^3^/s on 18th-20th September 1947 (see Fig. 1). The considerable difference between the peak discharges of the two river sections is caused by the retention in the channel and the bordering areas (Spreafico and Weingartner 2005).

**Fig. 2:**
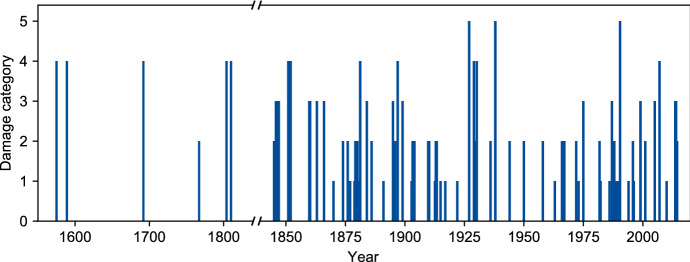
Flooding chronicle of the Gürbe River, 1575–2020

The discharge measurements already indicate the considerable flood hazard in the Gürbe valley: With an annual precipitation of over 2000 mm, the upper reaches belong to the area with the highest precipitation of Switzerland. During the summer months, there are often heavy thunderstorms, and daily precipitation of 40–50 mm is frequent (Schwarb et al. 2001). In combination with steep slopes, soft rock, and soils with low water storage capacity, these factors often lead to floods, landslides and debris flows (Fig. 2).

## Reconstructing the historical floods of the Gürbe River: data and method

Basic information about the historical floods is provided by the discharge measurements. They indicate the dimension of the event and show the exact date and time. However, measurements only provide limited information. The gauging data only show the maximum discharge, discharge, but not information about the flood's cause, extent, or damage. Additionally, discharge values are often distorted because the tools to gauge water levels are damaged or lose their measurements value once the water has left the riverbed. For estimating the retention in the valley floor, two gauging stations are needed—one in the upper and one in the lower reach. At the Gürbe River, this has only been the case since 1981.

This leads to a third problem of the discharge measurements: They rarely span more than a century (Hall et al. 2014). In Switzerland, gauging stations on lakes and large rivers were mostly installed during the nineteenth century, with a few already installed in the eighteenth century (Pfister 1999). However, small rivers were equipped with measurement instruments only during the twentieth century. Considering the changing climate in the past centuries but also in the present and the future, the short data sets are insufficient for chronicling floods and estimating extreme events. Therefore, it is essential to supplement the measurement series with historical information (cf. Himmelsbach 2014; Pfister 1999; Scherrer et al. 2011; Schulte et al. 2019). Different archives provide information on historic floods: historical, botanical, and geological archives have been recognised as valuable sources for estimating flood risk (Benito et al. 2004; Brázdil et al. 2006; Wilhelm et al. 2018). In the ideally case, data from different archives can be integrated. In reality, research often has to rely on datasets based on only one archive due to limited data availability.

For reconstructing the historical floods of the Gürbe River before 1800, as for many other small rivers and mountain torrents, only historical documents are available. Luckily, these sources are diverse and—at least for recent centuries—numerous: information on past flood events can, among others, be found in compilations of flood reports (e.g., from insurance companies), newspapers and journals, publications on local history, pictorial sources, or epigraphic sources such as watermarks (Brázdil et al. 2006; Glaser et al. 2010; Wilhelm et al. 2018). For reconstructing floods of the Middle Ages or Early Modern period, annals, chronicles, memorial books or memoirs and accounting books are crucial (Pfister 2018; Wetter 2017). One category of sources must be emphasized: the flood protection documents. These documents, consisting of technical reports on hydraulic structures, damage reports or subsidy applications, often contain useful information on the date, the course, the damage, or the cause of previous flooding events. They often also contain information on small or medium floods, which is generally much more difficult to find than information on the large events. The variety and concentration of source material and therefore the information density is higher the closer the event is to the present. Accordingly, information about floods further back in time is more incomplete and uncertain. With the aim of creating a flooding chronicle for the Gürbe River, I gathered all information discovered in the archives and public records, as well as through conversations and private holdings. They were contextualized and cross-validated with corroborating sources. The result is a highly detailed flood chronicle.

In order to compare the magnitude of flooding or the extent of damages or the climatic causes, different methods come into question. A promising approach which has lately been used for several Swiss rivers is the reconstruction and comparison of instrumental and pre-instrumental discharges (see Näf-Huber et al. 2016; Wetter et al. 2011; Wetter 2017). However, an application of this method is not possible for the Gürbe River since the riverbed is extremely variable due to the debris transport during floods which makes calculating the discharges problematic. Also, the sources required for this such as flood marks are lacking, which can be explained with the limited land use in the flood plain until the mid-nineteenth century (see discussion). Better suited for mountain torrents are comparison methods based on the damage and the spatial extent of the floods. Such index-based reconstructions are widely used, and they exist in different versions (c.f. Deutsch and Pörtge 2002; Glaser et al. 2002; Himmelsbach et al. 2015; Pfister and Hächler 1991; Rohr 2007). They have some disadvantages such as the lack of mathematical exactness, but also important advantages: they allow the utilisation of a wide range of historic sources and the inclusion of information about the spatial extent, the causes, or the damage. Furthermore, they reflect the fact that floods only find resonance by disturbing the daily routine—not every high discharge was recorded or even perceived.

It is important to keep in mind that the highest discharges do not automatically coincide with either the highest damage or the highest damage sums. The latter strongly increased in the nineteenth and twentieth century due to the—necessarily exposed—hydraulic structures and the intensification of the land use in the riverine zone in the late nineteenth and twentieth centuries (Kundzewicz et al. 2014). Also, since the 1950s, people have been storing increasingly expensive equipment in their cellars (Röthlisberger 1998).

This article applies Christian Pfister and Stefan Hächler’s classification scheme to the Gürbe River (Pfister and Hächler 1991). It consists of two steps. First, the damage is categorised with a four-point scale (Table 1). The scale is adapted for small rivers and mountain torrents by removing fatalities and taking the overbank sedimentation and the damage hydraulic structures into account.

**Table 1:** Damage categories, based on Pfister and Hächler (1991), adapted for small rivers and mountain torrents

Damage	Description
Little	Little damage to fields, gardens, and forest close to the river. Damage to infrastructures such as roads and footbridges. Minor damage to hydraulic structures. Local supporting and reconstruction measures, modest costs
Considerable	Medium damage to fields, gardens, forest, and infrastructure close to the river. Medium damage to hydraulic structures. Significant reconstruction costs
Heavy	Significant damage or partial destruction of the protection structures and infrastructure. Severe damage to fields, gardens, and forest, overbank sedimentation. Coordinated supporting measures, e.g., for reconstruction of hydraulic structures
Very heavy	Severe damage and large-scale destruction of flood protection structures. Severe damage to buildings and infrastructure. Severe and large-scale damage to fields, gardens, and forest. Destruction of a significant part of the harvest. Supra-regional supporting measures. Long-term reconstruction of hydraulic structures

In the second step, the damage category is combined with the spatial extent. Since the Gürbe River is only 30 km long, the scheme of Pfister and Hächler had to be adapted. Instead of the categories local/regional/supra-regional it now differentiates between 1 and 2 affected municipalities or 3 + . In total, 26 municipalities are located in the Gürbe valley. The result is a classification of the historical floods within the categories small/medium/severe/very severe/catastrophic (Table 2).

**Table 2:** Combination of the damage categories with the spatial extent. System based on Pfister and Hächler (1991), adapted for small rivers and mountain torrents

Spatial extent	Damage in 1–2 municipalities	Damage in 3 + municipalities
Little	Small (1)	Medium (2)
Considerable	Medium (2)	Severe (3)
Heavy	Severe (3)	Very severe (4)
Very heavy	Very severe (4)	Catastrophic (5)

A total of 78 historical floods of the Gürbe River could be found for the period from 1575 to 2020, of which 20 are categorised as small, 27 medium, 16 severe, 12 very severe, and 3 catastrophic. Unlike many of the large Swiss rivers, the Gürbe River was affected not only by small or medium, but also by severe and even catastrophic floods in the twentieth century. Accordingly, no disaster gap can be identified as is the case on larger study scales (c.f. Blöschl et al. 2020; Pfister 2009). The difference between the decades before 1800 and the last two hundred years is a problem with the data availability.

About two-thirds were caused by thunderstorms, a bit less than a quarter by long-lasting precipitation, and just ten percentage by a combination of precipitation and snowmelt. Most floods occurred during summer (seasonal distribution: DJF: 5, MAM: 7, JJA: 47, SON: 15, N/A: 4). This analysis reveals a dissimilarity in the flooding in the river's upper and lower reaches.

The upper reach had to bear the brunt regarding the frequency and the damage: 29 out of 78 flood events affected the full length of the river, 33 only the upper reach and 16 only the lower reach. The floods in the upper reach were mostly caused by thunderstorms and dominated by large amounts of bed load. Accordingly, the main damage occurred due to overbank sedimentation in the area where the slope lowers significantly. In the lower reach of the Gürbe River, floods were usually caused by longer precipitation events. The damage was usually due to water covering the ground for days. The entire Gürbe River was affected mostly by large-scale precipitation events that affected large parts of northern Switzerland and many parts of Western and Central Europe (e.g., in 1852, 1876, 1910 and 2005). The flooding chronicle points out that flood protection always was—and still is—an important issue at the Gürbe River as well as many rivers throughout Europe.

## Flood protection at the Gürbe River from 1800 until today

The flood protection history of the Gürbe River can be divided into five phases: the uncoordinated small-scale measures from the Middle Ages until the mid-nineteenth century, the 26 years of river regulation (so called “Grosse Gürbekorrektion”), the phase with an intense construction activity in the upper reach in the late nineteenth and early twentieth centuries, the decades in the middle of the twentieth century with numerous recreation and renewal projects and finally the 1990s and 2000s with a major paradigm change. The following section gives an overview over these five phases and shows in what regards the small river was typical and which developments were exceptional.

### Until the mid-nineteenth century: small-scale flood control measures

Until the mid-nineteenth century, the riverbed of the Gürbe River was wide and elevated due to the massive bed-load transport. In some stretches, the river was even at the same level as the surrounding areas, which was one of the reasons for the frequent flooding. In the transitional zone of the steep upper reach to the flat lower reach, the sediment deposit even had a width of 90 to 150 ms. The surrounding floodplain was dominated by swampland with reeds and alluvial forest (Vortrag an den kleinen Rath, 1811).

Old maps and regulation plans provide important sources for the reconstruction of the river's environmental conditions before regulation (Black 1997; Horst 2008; Schenk 2003). In some stretches the Gürbe River flowed as a braided river, in others it meandered. As was common practice in the Middle Ages and Early Modern times, the main flood protection strategy was to avoid hazardous areas (Vischer 2003). Only in areas where the locational advantages outweighed the disadvantages of the flood hazard or where were no other possibilities existed did people try to protect themselves and their properties. The early hydraulic engineering measures were mostly done in urban areas, where the water served many different purposes such as sanitation, transportation, power supply, fishing, or waste disposal. These water uses frequently required interventions in waterways which were often contrary to flood protection measures (Longoni and Wetter 2019). Landowners and users mostly carried out small-scale measures, often creating problems downstream (Schmidt 2000; Vischer 2003).

Only three settlements in the Gürbe valley were on the river: Belp near the river's mouth and Blumenstein and Wattenwil on the alluvial fan. Settling on alluvial cones was popular despite the considerable hazard of floods and debris flows since the soils were more fertile and less swampy than the ones in the valley floors, making them better not only for agriculture but also for building. To protect the houses and the arable land from the water, longitudinal and transverse structures such as sills were built (Egger 1958).

Except for the aforementioned villages, all other settlements and all major roads were situated on the flood-safe hillsides. The wet areas with poor-quality soils in the flood plain were used as common lands as was typical in traditional agriculture (Pfister 1995). Accordingly, people only minimally used the valley floor for activities like pastures, peat cutting or reed harvesting. Crops were only planted in elevated areas (Graffenried 1761). Despite the limited land use, some old maps and documentary sources indicate that riparian communities had already built small hydraulic structures to protect their fields from the Gürbe River. Wooden training structures, dams and fascines (bundles of brushwood used protecting the banks of streams from erosion) amended and stabilised the watercourse and sills made of timber and stone levelled the gradient. However, all early efforts were uncoordinated and therefore only effective for a short time (Bericht des Regierungsstatthalters, 26.11.1832). In the 1730s, people even straightened a longer river stretch with the intention of facilitating rafting (Hartmann 1752). Maps from later decades suggest that this straightening did not last. In the steep upper reaches, no hydraulic structures were built yet. Although check dams had been tested in Tyrol in the sixteenth century, the gorges of the European torrents generally remained unobstructed until the second half of the nineteenth century (Duile 1826; Schnitter 1992).

### 1855–1881: river regulation

After centuries with small-scale flood protection measures as the only option, the situation profoundly changed during the nineteenth century. Hydraulic engineers now regarded the watercourses as coherent systems and identified the rivers' large widths and low gradients in the floodplains as the main cause of the floods. Therefore, rivers should be consolidated, shortened, and the water should run off as straight and unobstructed as possible (Speich 2003; Vischer 2003). The change from small-scale to large-scale flood protection had several reasons. Science and technology (especially hydrology and hydraulics) had made major progress since the late eighteenth century (Vischer 2003). The engineers could now calculate the runoff of rivers, and new building materials and machines were available. Additionally, the perception of nature changed profoundly in the eighteenth and nineteenth century since the idea prevailed that people could control and shape nature (Walter 1996). Another reason was the rapid population growth which made the arable land scarce. More and more people—especially the lower classes—were forced to settle and cultivate their crops and vegetables in the flood prone areas (Protokoll Grosser Rath, 28.11.1854). The gain of new agricultural land was one of the main reasons for the demands for river regulation. The initiators of the Gürbe regulation argued that the food self-sufficiency could be improved by the drainage of 1800 hectares of agricultural land close to the Swiss capital, Bern. Fourthly, an accumulation of severe floods in western Europe intensified the demand for protection measures (Blöschl et al. 2020; Pfister 1999). In contrast to other watercourses, transport projects such as the navigability of rivers or railroads did not matter on the Gürbe River in the mid-nineteenth century.

The implementation of the river regulation and the torrent control was possible due to political changes in the Canton of Bern, where the Gürbe was located, during the first decades of the nineteenth century. In the context of political transformations and state-building processes, hydraulic engineering laws were enacted from the 1830s onwards. The canton started to subsidise hydraulic engineering projects and the implementation of large-scale projects therefore became feasible.

**Fig. 3:**
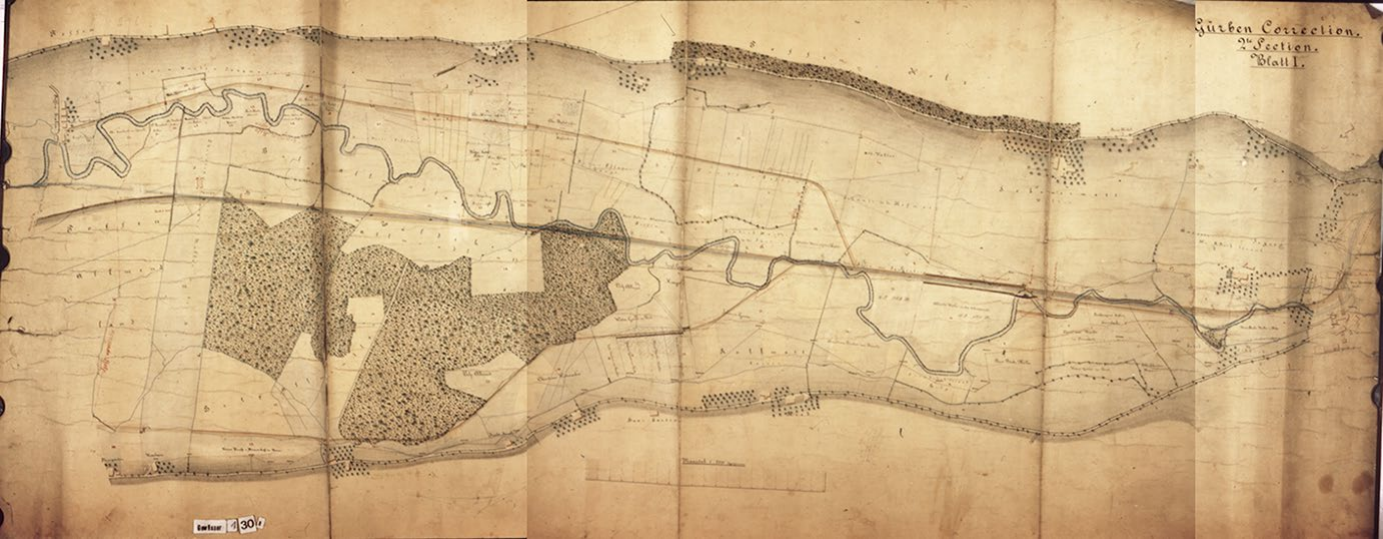
Map of the Gürbe River between Toffen and Belp, 1870. The map shows the old meandering course of the river and the new straight channel. State archive of the Canton of Bern, StAB AA V 130

The first attempts at a coordinated flood protection project of the Gürbe River had already been made in the early 1800s, the 1830s and the early 1840s by local residents, but they all failed for financial reasons (Salvisberg 2017). A petition, filed by dozens of landowners in 1846—a year with a severe flood—was finally successful. The Canton of Bern decided to support and co-finance the river correction. After some years of preparation (the Canton of Bern even passed a law for the project), the so called “Grosse Gürbekorrektion” was implemented from 1855 to 1881. The previously meandering Gürbe River was straightened and channelised (Fig. 3), as with most of the Swiss alpine rivers (Hohensinner et al. 2020).

**Fig. 4:**
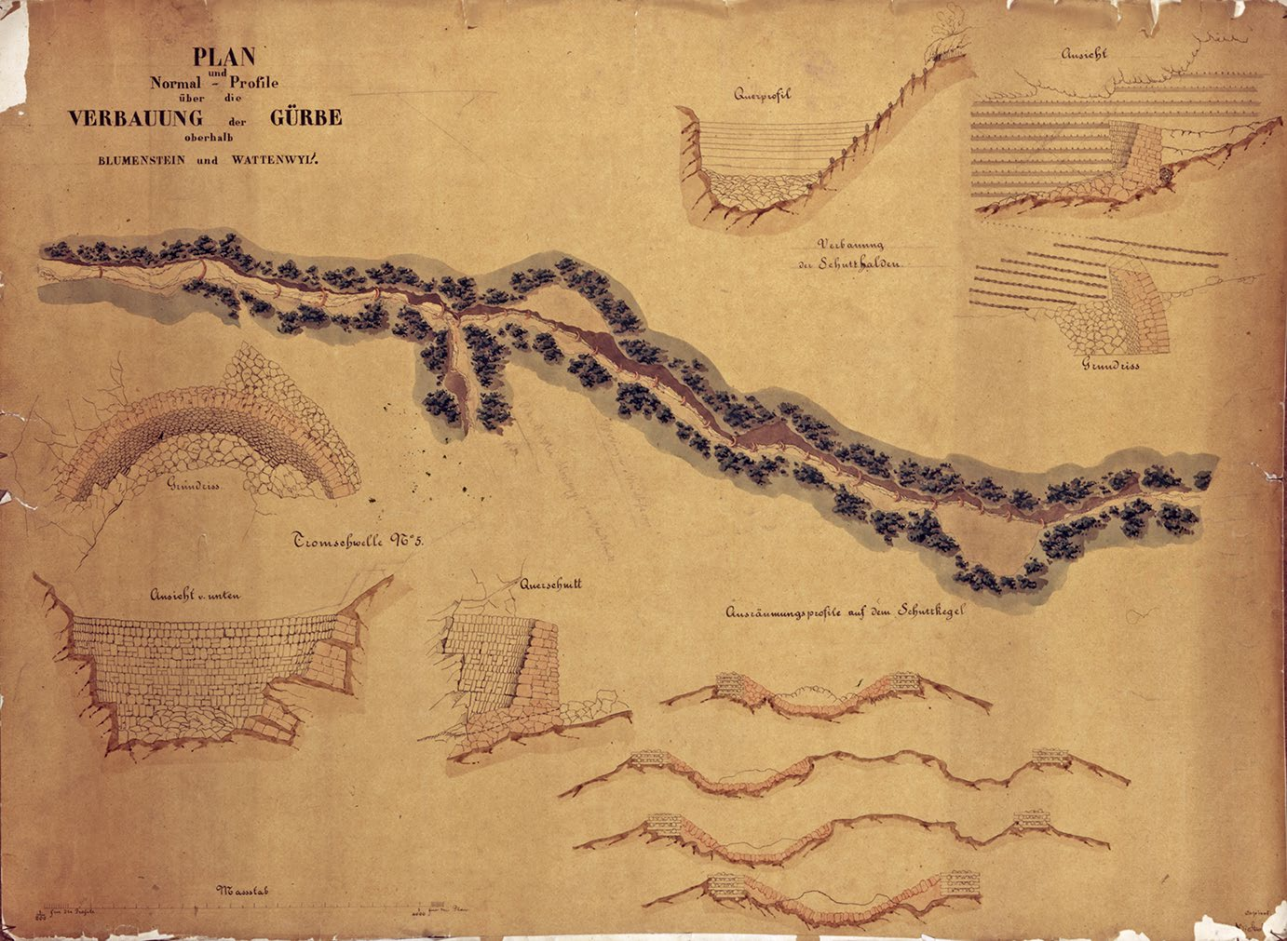
Overall plan and profiles for the construction of the check dams in the gorge of the Gürbe River, ca. 1860. State archive of the Canton of Bern, StAB AA V 116b

In the steep upper reaches, the construction workers raised a series of dams, built of logs and local stone, to limit the geomorphic activity (Hess 1876). Debris flow deflection dikes, drainage systems and slope stabilisations completed the system (Fig. 4). The series of check dams had been developed by the Tirolean Joseph Duile (see Duile 1826) and had until then only been implemented in a few mountain torrents in the Canton of Glarus in the 1840s. In the following decades, Duile’s technique spread internationally (Vischer 2003).

As was typical for the nineteenth century, hydraulic engineers planned and promised that the protective structures would prevent inundations completely (Hannig 2019; Verordnung über die Schatzung, 19.03.1855). At the Gürbe River, as on many other rivers, this goal was not achieved. Although modifying the river alleviated the flood situation, flooding was still frequent. The floods of 1860, 1866, 1870, 1874, 1879, 1880, and 1881 damaged or destroyed the new hydraulic structures and even the construction sides. Additionally, the bed load did not decrease as expected, preventing the completion of planned torrent control measures. When the federal state passed the first hydraulic engineering laws and started paying subsidies for torrent control, the subsequent organisational restructuring forced the “Grosse Gürbekorrektion” to end. All remaining protective measures would be conducted within the scope of new flood protection projects.

### 1882–1910: subsequent projects and focus on the torrent control

**Fig. 5:**
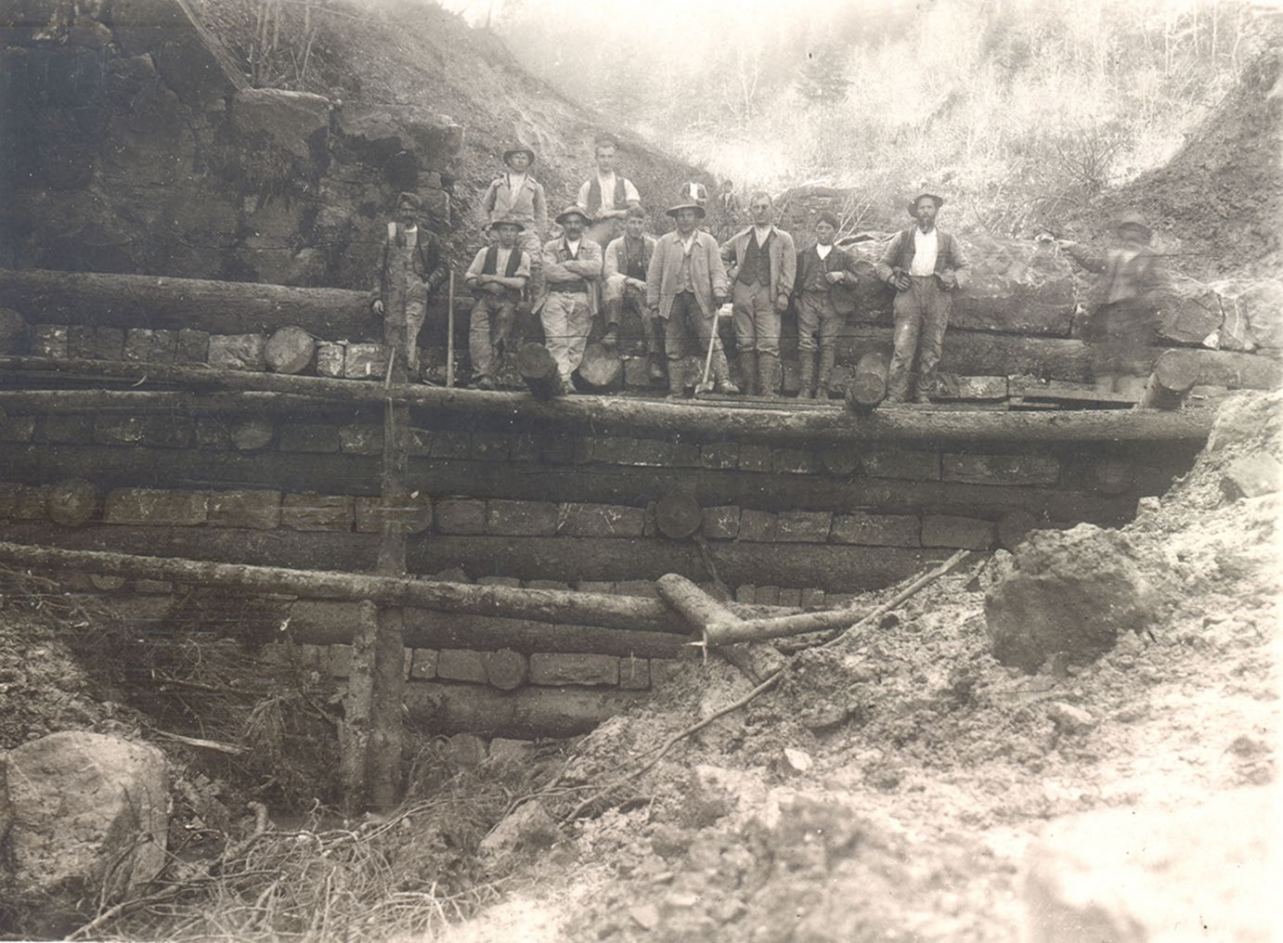
Construction workers raising check dams in the gorge of the Gürbe River, ca. 1900. Copyright: Lieselotte Kappeler

From 1882 onwards, the flood protection measures at the Gürbe River were implemented as subsequent projects to the “Grosse Gürbekorrektion”. The flood protection measures were normally financed one-third each by the canton, the federal state, and the landowners/municipalities. The focus lay mostly on the torrent stretch in the upper reach, which was typical for this period. In Switzerland as in other alpine regions such as Bayern or Tyrol, the torrents became the key concern in the late 19th and early twentieth centuries (see e. g., Göttle 1996; Piton et al. 2016; Schnitter 1992). At the Gürbe River, construction workers—now guest workers from northern Italy—expanded the torrent control system (Fig. 5). They raised additional and larger transverse structures not only in the gorge of the Gürbe River but also in several tributaries.

As a result, the protection system became not only bigger but also denser and larger. From 1900 onwards, the torrent check dams were built of concrete instead of stones and timber. The hydraulic engineers announced the prospects of success of the flood protection projects each time optimistically. Friedrich von Werdt, a government member of the Canton of Bern was sure that the Gürbe Valley would be safe from floods after the implementation of the project: “It is quite safe to assume that after the project has been carried out, the valley will no longer have to suffer much from the devastation of the Gürbe River.” (Protokoll Ausschuss Gürbekorrektion, 15.10.1892).^1^

In addition to the hydraulic engineering works, hundreds of hectares of alpine meadows were reforested in the catchment area of the river (Nigst 1914). This was linked to the newly widespread explanation of deforestations in the upper reaches as being responsible for the floods in the valley floors (Culmann 1864; Landolt 1862). French engineers had first made this connection in the late eighteenth century. In Switzerland, by lobbying in the mid-nineteenth century, the Swiss Forestry Society contributed significantly to the success of this idea (Pfister and Brändli 1999). As with many other parts of pre-Alpine areas, the catchment area of the Gürbe River had mostly been deforested in the early nineteenth century. Therefore, the Canton of Bern and the federal state demanded an afforestation as a condition for the subsidies. However, several owners of the alpine meadows refused to provide their land for the afforestation projects. This caused conflicts that persisted for decades (Salvisberg 2017).

In addition to the upper reach, the flood protection at the lower reach turned out to be difficult as well. The fields and settlements were still flooded frequently. Consequently, the still naturallyflowing stretches of the Gürbe River in the transition zone and the river mouth were channelised. In the beginning of the twentieth century, the Gürbe River was finally embanked and consolidated from its mouth to its source.

Nevertheless, the channel already had to be re-engineered; recurring floods proved that the channel width was too narrow. While engineers had calculated peak discharges of 41 m^3^/s in the upper reach and 59 m^3^/s in the lower reach, they had to adjust the estimation. After the floods of the 1880s, plans acknowledged that discharge up to 90 m^3^/s could occur. Furthermore, the maintenance of the flood protection structures turned out to be labour intensive, even more so since the transportation of debris had not yet stopped (Bericht und Kostenanschlag 1892).

### 1911–1990: ongoing enlargement and renewal

For nearly the entire twentieth century, the flood protection cooperatives and the cantonal hydraulic engineers focused on achieving the—so far failed—19th-century plans. Since landslides and floods still led to damage, they renewed and expanded the hydraulic structures in numerous subsequent projects. In the eight decades between 1911 and 1990, a total of 44 hydraulic engineering projects were implemented on the Gürbe River and its tributaries (see Salvisberg 2017). The flood protection philosophy had not yet changed. The engineers as well as the local residents still expected the hydraulic structures to prevent the floods completely. Since this goal was obviously not achieved with the existing structures, the flood protection cooperatives enlarged and extended the existing constructions. In addition, more and more hydraulic structures were raised in the tributaries. Thus, the flood protection system grew still larger and larger.

Particularly difficult decades were the 1920s and 1930s, when several very severe or catastrophic floods happened within a short period of time (1927, 1929, 1930, and 1938). The disasters were so frequent that flood protection cooperatives were not even able to repair the damaged hydraulic structures before the next event happened. Floods showed the deficiencies of the existing protection structures. Within the framework of the reconstruction works they not only rebuilt the hydraulic structures but also improved and extended them since they explained the floods by the incompleteness of the protection structures. In addition, efforts in reforesting were intensified since the flood protection experts acknowledged the still unfinished reforestations as a main cause of the flood events (Das Eidgenössiche Departement des Innern, 11.07.1928). They still did not discuss the problems of the prevailing flood protection philosophy yet (Summermatter 2012).

Throughout the entire twentieth century, but especially during the interwar period, the financing of the flood protection projects proved to be a major difficulty. There were two main reasons for this: firstly, Switzerland went through an economic crisis in the late 1920s and 1930s (see e.g., Müller and Woitek 2012). Secondly, landowners were confronted with a double burden since they not only had to pay for the flood protection measures but also for the ameliorations that were implemented during this time. The aim of these projects was the improvement of the agricultural conditions, since the river’s regulation in the nineteenth century had not led to the expected success concerning the drainage of the valley floor. The ameliorations not only included improvement of the soil condition, but also the land redistribution and the construction of agricultural access roads (Forrer 1952; Leuenberger 1935). Such ameliorations were implemented in most parts of Switzerland during these decades (Auderset et al. 2018; Stuber and Bürgi 2018). The financial double burden weighed heavy on the farmers, and some could not pay their share and got into debt (Leuenberger 1935). Like their members, the flood protection cooperatives also ran into financial difficulties in the 1920s and 1930s. In response, the cooperatives expanded the perimeter—the geographical boundaries of who needed to contribute to funding for hydraulic works. In addition to the ongoing projects and never-ending maintenance activities, renovation works became necessary from the 1960s onwards because the hydraulic structures were out-dated (Salvisberg 2017).

**Fig. 6:**
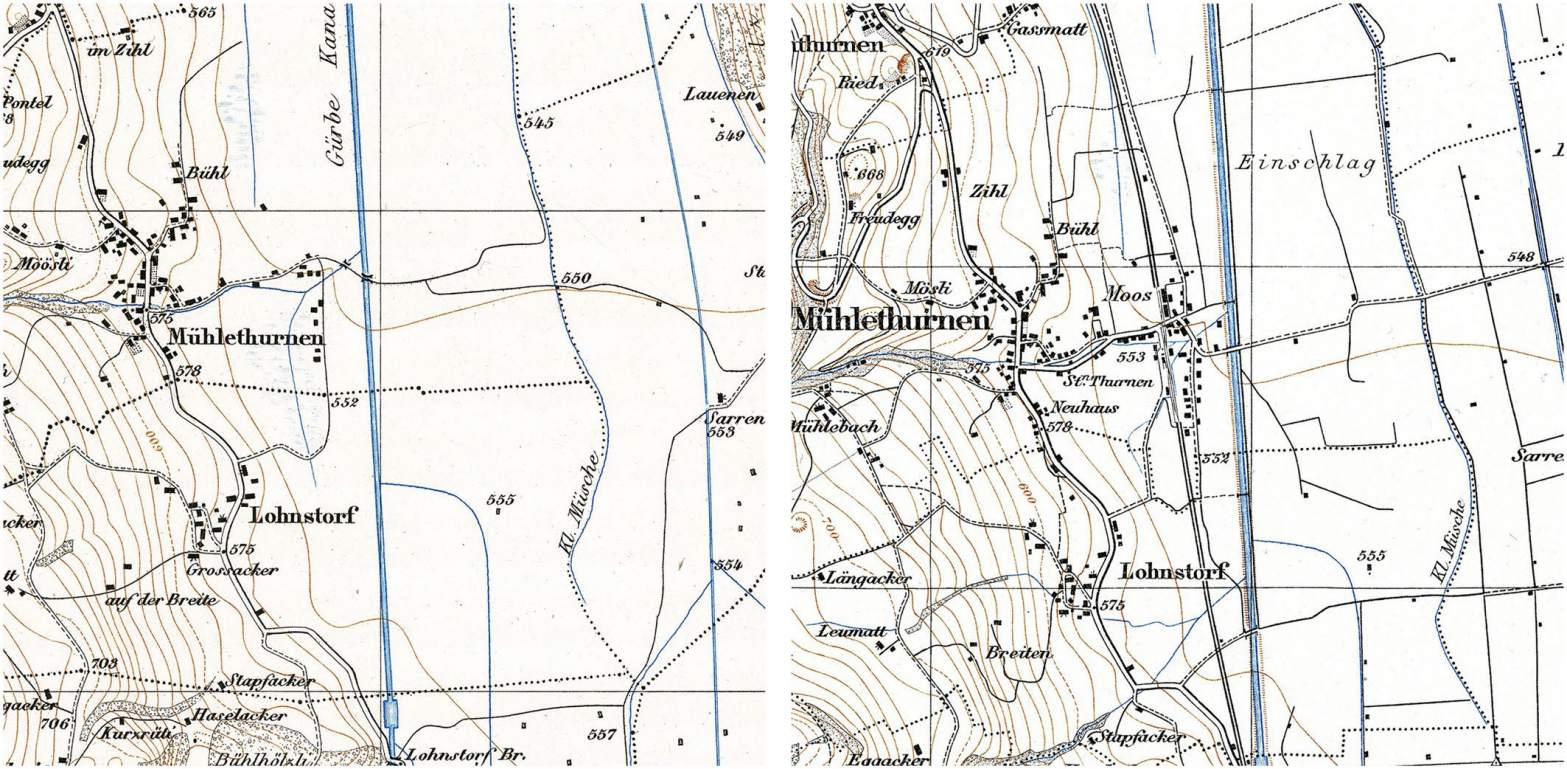
Siegfried Map, section Mühlethurnen, editions 1873 and 1939. The blue shading shows the swamps. In 1873 (left), only few small roads were located in the hazard zone. In 1939, the agricultural land had been ameliorated and several linear access roads had been built. The airport Bern-Belp, opened in 1929, is located directly next to the Gürbe River. Copyright: swisstopo

In connection with flood protection, land use practices in the riverine zone transformed fundamentally. Similar developments can be seen on many other rivers; on small ones such as the Limpbach (Bürgi et al. 2010) or large ones such as the Danube (Hohensinner et al. 2013; Winiwarter et al. 2013). Based on the assumption that the valley floor was (or soon would be) safe from flooding, people started to use the floodplain more and more. For agricultural development, the flood protection and especially the subsequent land improvement projects were a great success. People started to plant crops and vegetables on the newly won farmland up to the riverbanks. A total of 1800 hectares of new agricultural land were gained (Forrer 1952; Hess 1876). In addition, houses, roads and a railroad could be built on the flat valley floor. This development is visible on the “Siegfried Map”, the official map series of Switzerland from 1870 to 1926 (updated until 1949). The map section of the village of Mühlethurnen shown in figure 6 documents these changes.

In 1873 the houses were still predominantly on the flood-safe on the hillsides away from flood zones, and only a few buildings stood along the small roads. By1939, things had changed: a railway line ran close to the Gürbe channel, and the roads were modernised and enlarged. In the conveniently situated area of the railway station (which is very close to the river), a second village centre had been built (Fig. 6). After the Second World War, the settled area grew and spread further into the valley floor. Today, a total of 818 buildings are located in the floodplain of the Gürbe River, an increase of 210% compared to the early nineteenth century (Zischg 2016).

Maps, as with many other sources, prove that land use in the riverine zone became more and more intense over the decades. This development occurred parallel to the enlargement of the hydraulic structures, though it also created new vulnerabilities. As engineers constructed more protective structures and land near the river thus appeared safer, more and more people and businesses settled along the river, gradually increasing need for more flood protective measures. This connection was not recognised or questioned for decades.

### 1990–today: restorations and difficult revitalisations

After decades of persistence, the last years of the twentieth century witnessed a period of change caused by the reevaluation of flood protection that took place in Switzerland as well as in many other countries (see Adams et al. 2004; Bebermeier 2011; Johnson et al. 2005; Kruse 2010; Lübken 2014; Muhar et al. 2009; van der Werff 2004). In Switzerland, reevaluation began in the 1960s among engineers of the federal state and the canton, spurred by increasing environmental awareness (Summermatter 2012). In the years that followed, they gradually reviewed the principal goals of flood control. The protection concept changed from technology-oriented to nature-orientated: engineers sought to consider ecological consequences when modifying watercourses and use so-called “green infrastructure” to protect against flooding. Engineers and planners have begun to prioritize passive protection measures wherever possible (Götz 2003; Zaugg Stern 2006). The different requirements such as flood protection, water conservation or recreational use were now better coordinated (Bernhardt 2016).

In Switzerland, the new principles were included in guidelines and ultimately in the laws from the 1960s onwards (federal law in 1991). However, at the local level of the Gürbe River, reevaluation did not occur contemporaneously. The engineers and flood protection cooperatives hesitantly discussed the new principles from the 1980s but did not implement them yet. Two natural disasters finally triggered the change. The first one was a landslide and debris flow in the Tiefengraben in the upper catchment area of the Gürbe River in April 1987. The disaster was caused by a thunderstorm that coincided with the snowmelt and therefore heavily saturated soils, approximately 40,000 m^3^ of soil, debris and wood slid down (Kellerhals and Haefeli, 24.11.1987). The debris flow caused heavy damage. To plan the reconstruction projects, the flood protection cooperative of the upper Gürbe River launched a study about the causes of the event and the future of disaster management in this area (Integralbericht 1991).

Before the study was finished, another catastrophic flood happened on 29 July 1990. After a warm and sunny day, a huge thunderstorm appeared in the upper reach of the Gantrisch region. The pluviograph measured 240 mm precipitation after only three hours, which was a record. Within a short period, the water level of the Gürbe River and its tributaries rose rapidly. The water and especially the bed load caused enormous damage to hydraulic structures, settlements, transport infrastructure, agricultural land and forests. The inspection after the event was disheartening since large parts of the flood protection structures in the upper reach were destructed. Out of a total of 140, 84 check dams had collapsed or were heavily damaged and therefore ineffective, and important sections of the deflection dikes had collapsed. In some parts, the river was hardly recognisable since the water had carved new paths and the river bottom had lowered massively. In the transition zone between the steep upper and flat lower reach the water had left its bed and flooded the surrounding areas. About 200,000 km^3^ of bed load were deposited in Wattenwil and Blumenstein. The damage on the hydraulic structures was 35 million Swiss Francs (Protokoll Geschäftsprüfungskommission, 24.08.1995). The upper reach had borne the brunt of the damages, but the lower reach was affected as well. The water, stones, wood and mud covered large areas of the agricultural fields. Several bridges were destroyed, and the railway service was interrupted for days.

The extent of the flood's damages was a shock, as so much had been done for almost a century and a half to prevent such events. Not only had the river been consolidated and embanked from its mouth to its source, but a total of 1800 hectares had also been reforested since the late nineteenth century. In 1990 55.6% of the upper catchment area was covered by forest, more than the intended 40% (BWG 2004). The events made it clear that flooding could not be prevented completely—no matter how large the torrent control and the protection forests were—and showed the need for a new protection concept. The maxim of preventing the formation of sediment and draining the water as fast as possible lost its validity. These two events thus accelerated the transition process that had started in the 1980s. Afterwards, not only the experts but also the broader public discussed if and how the hydraulic structures should be rebuilt (Integralbericht 1991).

The involved parties (flood protection cooperatives as well as cantonal and federal engineers and decision makers) finally decided to rebuild most of the torrent control since this was the only option if the settlement areas were to remain in place. They also decided that retention areas should be created, and the riverbed widened wherever possible. In addition, new requirements regarding land use and protection goals were defined by the cantonal engineers in collaboration with the flood protection cooperatives. For example, the hydraulic structures should protect settlement areas and important infrastructure such as the airport better than agricultural land (Salvisberg 2017).

In the years that followed, the flood control cooperatives implemented several large-scale flood protection projects. These projects not only included large new check dams and debris flow detection dikes but also a rake to contain driftwood. This structure—a pioneer in the 1990s—successfully prevents log jams on bridges in the lower reach.

The implementation of the nature-orientated protection measures required by the hydraulic engineering laws proved to be difficult. One main difficulty was the acquisition of the land since some of the landowners did not want to give up the land. They argued that the achievements of their ancestors had drained and cultivated the land. Other involved parties in these conflicts were the nature protection organisations, the anglers, the drinking water supplier, and the people that used the Gürbe River and its surrounding for recreation. All these interests needed to be considered and long-lasting negotiation processes were necessary.

In the upper reach, the possibilities were limited due to the geological conditions and the intense land use in the transitional zone. Therefore, engineers had to find compromises. Although some of the smaller structures could be created of natural materials such as local wood and stones, building workers rebuilt most check dams with reinforced concrete. The river stretches with less-steep slopes had more potential for extended renaturations. However, the implementation turned out to be challenging since the riverine zone of the Gürbe River was by now intensively used. Conflicts of interest arose that delayed the implementation of the projects for years. In 2021, 31 years after the flood, only two stretches of the Gürbe River have been revitalised. Some projects are currently being implemented, and others are still pending. Recent natural disasters, such as a 2018 landslide, again revealed the limits of both old and new protection measures. Debates about a further reshaping of the Gürbe River are still ongoing.

## Conclusion

The overview of the flood protection at the Gürbe River has demonstrated how much effort the landowners, the municipalities, the Canton of Bern, and the federal state of Switzerland have put into the flood safety of the Gürbe Valley. Since 1855, a total of 67 flood protection projects have been implemented, as have numerous smaller protection measures and continual maintenance. The stabilisation of the torrent section in the upper reaches in particular proved to be extremely difficult.

A retrospective analysis indicates that most of the projects were initiated in the aftermath of a flooding. Developments, such as technical breakthroughs, rarely triggered new projects, however, they were usually included in new projects that floods themselves had triggered. Not only severe or catastrophic floods but also small ones led to action, especially whenever they occurred frequently. Flood events made reconstruction works necessary, but they also called the flood hazard to mind. During reconstruction, planners often enlarged and improved the dams and dikes. This work was closely connected to the expectations for the protection measures. During the 19th and most parts of the twentieth century, all involved parties—the hydraulic engineers, the lawmakers, the local flood protection cooperatives, and the landowners—expected complete flood safety. Although the recurring floods disproved these promises, the expectations and flood protection philosophy proved to be persistent.

It was only in the late twentieth century that flood protection philosophy began to change, and the expectations became more differentiated. The protection against flood damage was still the primary aim given the more intensive use of threatened floodplains. In addition, other needs such as the ecological improvement or the recreational use of the Gürbe River should also be considered. However, new constructions were no longer intended to prevent the floods completely, but only to prevent serious damage. Today we are aware that flood damage cannot be wholly prevented, especially with mountain torrents. Residual risks should be recalculated and accepted. Therefore, torrent control systems can never be completed. Reevaluation first took place among the experts, and local communities have gradually also realized this fact, but this process is still ongoing.

The investment into the flood safety was immense was immense: the 'Grosse Gürbekorrektion' alone cost 2.9 million Swiss Francs (172 million in 2009) while the 1892 flood protection project was nearly CHF 750,000 (more than 36 million) (see Salvisberg 2017). Today the Gürbe River is known as one of the most expensive torrents in Switzerland, cost allocation was and remains one of the main sources of conflict for these projects. In some cases, disputes even had to be settled in court. The distribution of costs between the lower and upper reaches in particular caused conflict. The landowners and communities of the lower reach had to pay for construction in the upper reaches of the torrent because this infrastructure protected them. This cost allocation was repeatedly the cause of disagreement. Raising the money was often a challenge, especially for the landowners that had to finance the meliorations (e.g., Schwellenkommission Mittlere Gürbe an den Regierungsrat, 14.12.1938; Stürler 1959).

Knowledge about historical floods is important for many reasons: it helps to better understand hydrological processes and is crucial for planning hydraulic measures. In addition, it helps to raise awareness of flood risk in society and supports the current shift away from a complete promise of complete protection—which prevailed from the 19th to well into the twentieth century—towards a realistic and responsible approach to flood risk. This is an important issue on the Gürbe River because the watercourse is extremely problematic regarding flood protection. The frequent heavy precipitation in the upper catchment area causes a rapid rise of runoff. In combination with the steep slope in the upper reaches, the prevailing soils and the geological conditions, floods are frequent and cause considerable damage.

Since the riverine zone was used by humans from early on—as is typical for the densely populated Swiss pre-alpine areas—flood protection was already a concern of the riparian communities in the Early Modern era. Over the centuries, this development intensified. The regulation of the watercourse from 1855 onwards and the resulting cultivation of the valley floor led to an additional increase in intensity of use. A vicious circle developed: the more protective structures were built, and the better the land was protected, the more necessary and profitable flood prevention became. In the nineteenth century and large parts of the twentieth century the expectation emerged that floods could be prevented completely, which led to a steady expansion of hydraulic structures. Huge sums of money were invested in flood protection on this small river.

In the early twentieth century, the Gürbe River was embanked and consolidated from its mouth to its source. Additionally, large parts of the alpine meadows in the upper reaches were reforested.

It was not until the second half of the twentieth century that the principles of flood control gradually changed, though new principles arrived belatedly along the Gurbe River. The old maxim that the formation of bed load should be stopped became less important. Even though protection against serious flood damage remained the goal of the projects and serious damage was still to be prevented, residual risks were finally accepted. Furthermore, wherever possible, the engineers planed measures to improve the river’s ecological standing. A catastrophic flood in 1987 fostered the reevaluation on the local level. Nevertheless, the implementation of the new “green” flood protection measures proved to be very difficult due to the intense use of the watercourse area and the many conflicting interests.

## Data Availability

Not applicable.

## Footnotes

1 English translation: “It is quite safe to assume that after the implementation of the project the valley will no longer have to suffer from the floods of the Gürbe River.”
